# Engineering Extracellular Vesicles to Target Pancreatic Tissue *In Vivo*

**DOI:** 10.7150/ntno.54879

**Published:** 2021-04-15

**Authors:** Hiroaki Komuro, Yuki Kawai-Harada, Shakhlo Aminova, Nathaniel Pascual, Anshu Malik, Christopher H. Contag, Masako Harada

**Affiliations:** 1 Department of Cardiovascular Medicine, Tokyo Medical and Dental University, Tokyo, Japan.; 2 Institute for Quantitative Health Science and Engineering (IQ), Michigan State University, Michigan, USA.; 3 Lyman Briggs College, Michigan State University, Michigan, USA.; 4 Department of Chemical Engineering and Material, Michigan State University, Michigan, USA.; 5 Department of Microbiology and Molecular Genetics, Michigan State University, Michigan, USA.; 6 Department of Biomedical Engineering, Michigan State University, Michigan, USA.

**Keywords:** Extracellular Vesicles, Pancreatic β-cells, EVs engineering, Imaging, Targeted delivery.

## Abstract

Extracellular vesicles (EVs) are naturally released, cell-derived vesicles that mediate intracellular communication, in part, by transferring genetic information and, thus, have the potential to be modified for use as a therapeutic gene or drug delivery vehicle. Advances in EV engineering suggest that directed delivery can be accomplished via surface alterations. Here we assess enriched delivery of engineered EVs displaying an organ targeting peptide specific to the pancreas. We first characterized the size, morphology, and surface markers of engineered EVs that were decorated with a recombinant protein specific to pancreatic β-cells. This β-cell-specific recombinant protein consists of the peptide p88 fused to the EV-binding domain of lactadherin (C1C2). These engineered EVs, p88-EVs, specifically bound to pancreatic β-cells in culture and transferred encapsulated plasmid DNA (pDNA) as early as in 10 min suggesting that the internalization of peptide-bearing EVs is a rapid process. Biodistribution of p88-EVs administrated intravenously into mice showed an altered pattern of EV localization and improved DNA delivery to the pancreas relative to control EVs, as well as an accumulation of targeting EVs to the pancreas using luciferase activity as a readout. These findings demonstrate that systemic administration of engineered EVs can efficiently deliver their cargo as gene carriers to targeted organs in live animals.

## Introduction

Over the last two decades, studies of extracellular vesicles (EVs) have shifted our understanding of their role in biology from early classifications of EVs as waste disposal machinery to a role in intracellular communication 1. Cells secrete heterogeneous populations of lipid-bilayer membranous nano-sized particles such as exosomes, microvesicles (MVs) and apoptotic bodies whose composition may vary depending on the cell of origin, physiological and pathological condition of the cells or surrounding tissues 2. While the sizes of these particles largely overlap with each other, the differences are consistent with the distinctive biogenesis of exosomes and MVs. Exosomes (40-150 nm in diameter) derive from the inward budding of endosomal multivesicular bodies (MVBs) and are released from the cell upon MVB fusion with the cell membrane. MVs (50-1,000 nm in diameter) are generally larger vesicles and are the product of direct budding from the plasma membrane 3, 4. EVs are released from many different cell types into various body fluids, including milk, saliva, sweat and plasma, to mediate molecular transfer to other cell types in both physiological and pathological conditions 5-9.

A growing number of reports suggest small EVs (50 to 200 nm in diameter) facilitate the functional transfer of genetic material involved in diseases like diabetes, making EVs an appealing therapeutic gene delivery vehicle for immune therapy, vaccines, and regenerative medicine 9-20. In fact, EV-mediated gene delivery circumvents the significant issues associated with synthetic nanoparticles, such as instability, immunogenicity, toxicity and biological barrier crossing 1, 21-24. Naive EVs derived from stem cells or mesenchymal stem cells (MSCs) have ant-inflammatory, organ-protective and regeneration promoting properties, thus exert therapeutic effects in inflammation and organ injury 25-27. Furthermore, several groups reported EV-mediated therapeutic delivery *in vivo* 28-31. However, an EV engineering platform that can be readily modified for various cellular and tissue targets is essential for rapid development of EV-based therapeutics with clinical applications. Such a platform should be designed to overcome technical limitations, including a lack of controlled generation of EVs, inefficient loading, and inadequate tissue specificity 32-34. EV surface proteins contribute to EVs' natural tropism, hence, the modification of these proteins to include surface adhesion molecules and ligands, may direct specific binding to desired cells, tissues or organs 22, 30, 35-38. There are several methods proposed for ligand display on EV surface. One such method fuses targeting moieties to known EV membrane proteins such as Lamp2b and CD63 31, 39-41. Alternatively, the C1C2 domain of the phosphatidylserine (PS) binding protein milk fat globule-EFG factor 8 (MFG-E8), also known as lactadherin, enables versatile EV surface display since it can be used to decorate the EV surface when expressed from producer cells, or when added to purified EVs 42. Several groups have shown that lactadherin C1C2 can modify exosome surfaces and target fusion proteins, such as anti-EGFR nanobody, carcinoembryonic antigen and HER2 antibody, to EVs 22, 38, 43-45. It has been demonstrated that producer cells, transfected with an expression plasmid, package pDNA into EVs, and if that plasmid encodes a surface protein that is expressed on EVs then the EVs will contain the DNA representing the surface expressed protein 20, 46, 47. EVs from cells containing such pDNAs would then have a feature of bacteriophages that make phage display screens possible. Towards developing an *in vivo* EV display screening protocol, we evaluated tissue targeting and EV packaging in this study.

Here, we used C1C2 to display p88, a peptide with known affinity for the human and mouse membrane small ion transport regulator (FXYD2)γa, to generate pancreas targeting EVs. A contrast agent (CA) consisting of p88 with a chelator and GdCl3 showed a potential β-cell specificity as an imaging agent for the MRI (magnetic resonance imaging), suggesting p88 as a potential β-cell targeting peptide 48, 49. We demonstrate that β-cell targeting EVs can enrich pDNA delivery to the pancreas in living mice. The pancreas is an inaccessible organ for both surgical and drug treatment due to its anatomical position, and potential experimental drugs are not clinically applicable due to the complications derived from the toxicity or inefficient delivery 50-52. This study proposes a unique technique for EV-mediated targeted delivery to pancreatic tissue and supports the development of customizable EV-based targeted delivery vehicles for nucleic acid-based therapeutics.

## Materials and Methods

### EV-peptide display plasmid construction

**S**eamless **Li**gation **C**loning **E**xtract (SLiCE) mediates *in vitro* DNA assembly through a RecA-independent recombination mechanism between DNA fragments with short homologous ends 53, 54. The SLiCE reagent, a bacterial lysate, was prepared from *E.coli* DH5α strain and used for all the cloning in this work following a previously described procedure 53, 54. Briefly, EV-display constructs were created by SLiCE assembly of PCR fragments into pcDNA6.0 V5/His (Invitrogen) digested with *NheI* and *AgeI.* The signal peptide and lactadherin C1C2 domain were amplified from psd44-Lactadherin46 (a gift from Agnese Mariotti Addgene, plasmid # 46830) using the primer sets (Table S1) which included overhangs. The coding regions of mCherry fluorescent protein and the gaussia-luciferase (gLuc) bioluminescent protein were amplified from pLM-CMV-R-Cre 55 (Addgene plasmid #27546) and pcDNA3.1(+)-GLuc (a gift from Contag lab), respectively. The PCR fragment was amplified from the pcDNA-mCherry-C1C2 plasmid and assembled with the synthetic double-stranded oligonucleotide consisting of pep1 coding sequence and the (GGGGS)_3_ linker sequence 56 (pep1-3xG4S-C1C2) to generate pep1-EV display construct. Similarly, a fragment was amplified from the pep1-EV display construct and assembled from synthetic oligonucleotides consisting of p88 coding sequence with homologous ends (P885-3-1 and P885-3-2) to create p88-EV display construct. pcDNA backbone for both pep1 and p88 was further down-sized by removing unnecessary sequences including the mammalian selectable marker (Blasticidin) and the phage origin of replication through the single-piece SLiCE reaction of the PCR fragments. This resulted in pcS-p88-C1C2 and pcS-pep1-C1C2. Another fragment was amplified from the pcS-p88-C1C2 using a primer set (HA-3xG4S-F and HA-R) and assembled to generate Non-Peptide display construct, pcS-NP-C1C2. The primers and oligos are listed in Table S1. Each construct was purified using the QIAprep Miniprep kit (QIAGEN) and sequenced for validation. The Plasmid Plus Midi Kit (QIAGEN) was used for large scale plasmid preparation.

### Cell culture and treatment

The following cell lines, obtained from American Type Culture Collection (ATCC), were tested for mycoplasma: 293T (Human Embryonic Kidney cell line), NIT-1 (Mouse pancreatic β-cell line) and 4T1 (murine mammary carcinoma cell line). The cells were cultured in high-glucose DMEM (Gibco) for 293T or RPMI-1640 (Lonza) for 4T1 media supplemented with 100U/mL penicillin, 100µg/mL streptomycin and 10% (v/v) fetal bovine serum (FBS, Gibco). FBS was ultracentrifuged in PET Thin-Walled ultracentrifuge tubes (Thermo Scientific 75000471) at 100,000g with a Sorvall WX+ Ultracentrifuge equipped with an AH-629 rotor (k factor = 242.0) for 18 h at 4 °C to remove the bovine EVs and create EV-depleted FBS for use in the culture media for preparation of engineered EVs. NIT-1 cells were cultured in F12 (Mediatech Inc.) media supplemented with 100U/mL penicillin, 100 µg/mL streptomycin and 10% (v/v) fetal bovine serum (Gibco). All cells were maintained in a humidified incubator with 5% CO_2_ at 37 °C. For EV production, EV-display constructs were either transfected alone or along with an imaging EV-display plasmid into 293T cells. In-house PEI (polyethylenimine, Sigma 408727) transfection reagent was used, which works similarly to commercially available polymer- or liposome-mediated *in vitro* transfection reagents 57. Cells were seeded at 2x10^6^ in a 10cm cell culture dish for 24 h in regular culture media and transfected with 10 µg total DNA suspended with PEI in non-supplemented DMEM. To prepare the DNA-PEI transfection mixture, 10 µg DNA/100 mm dish was added to PEI in a ratio of 1:2.5 (DNA:PEI) in non-supplemented DMEM, pulse-vortexed for 30 sec, and incubated at room-temperature for 10 min. Following 24 h incubation, cells were washed twice with PBS, and the culture media was replaced with DMEM supplemented with EV-depleted-FBS for another 24 h incubation for EV production. For naïve EV production, cells were cultured with DMEM supplemented with EV-depleted FBS without transfection for 24 h. mCherry labeled and p88-mCherry co-labelled EVs were prepared from 293T cells transfected with the mCherry-EV display construct (pcDNA-mCherry-C1C2) and co-transfected with mCherry-EV display and p88-EV display constructs. EVs labeled with gaussia-Luciferase (gLuc), co-labelled with p88-gLuc, co-labelled with PEP1-gLuc and co-labeled with non-peptide (NP)-gLuc were prepared by transfecting 293T with plasmid pcD-gLuc-C1C2 and co-transfection with pcS-p88-C1C2 58*.*

### EVs isolation

The cells were grown in DMEM media supplemented with EV-depleted FBS for 24 h, and the media from the plates was collected. For each batch, EVs were purified from 36 mL of conditioned media by differential centrifugation. The media was centrifuged at 400g for 10 min and then 600g for 30 min to remove the cell and cell debris. In order to remove the contaminating apoptotic bodies, the media was centrifuged at 2000g for 30 min. The supernatant was then ultracentrifuged in PET Thin-Walled ultracentrifuge tubes (Thermo Scientific 75000471) at 100,000g with a Sorvall WX+ Ultracentrifuge equipped with an AH-629 rotor (k factor = 242.0) for 90 min at 4 °C to pellet the EVs 59. The pellet containing EVs was resuspended in 100 µL PBS or PBS with 1% trehalose 60 except the gLuc labeled EVs, which were resuspended in DPBS (PBS with calcium and magnesium; Gibco 14190136).

### Nanoparticle Tracking Analysis (NTA)

The particle size and concentration were measured using a ZetaView® (Particle Metrix) Nanoparticle Tracking Analyzer following the manufacturer's instruction. EVs were diluted in PBS between 100- and 1000-fold to obtain a concentration within the recommended measurement range (0.5X10^5^ to 10^10^ cm^-3^).

### Western Blotting

Cells (transfected and non-transfected) were lysed by MRIPA lysis buffer (150 mM sodium chloride, 1.0% Triton X-100, 0.25% sodium deoxycholate, 50 mM Tris, pH 7.4), and the supernatant was used as cell lysates. Protein concentration was measured by Micro BCA Protein Assay kit (G Biosciences) using BSA as a standard. 50 µg of the protein and 1x10^9^ EVs were denatured at 70°C for 10 min in NuPAGE LDS Sample Buffer (Thermo Fisher Scientific), separated on a 12% SDS PAGE, and transferred to a nitrocellulose membrane. The membrane with the blotted proteins was blocked with blocking buffer containing 5% milk in Tris-buffered saline (TBS) for 2 h and then incubated with a primary antibody at 4°C overnight. Following three washes with TBS with 0.1% Tween 20 (TBST), the membrane was incubated with secondary horseradish peroxidase-conjugated secondary antibody for 1.5h at room temperature. The membrane was again washed thrice with TBST and the protein bands were visualized by treating with SuperSignal West Pico PLUS chemiluminescent substrate (Thermo Scientific) and the image was captured by ChemiDoc Imaging System (BioRad). The following primary antibodies were used: anti-HA (Sigma Aldrich, H3663), anti-β-actin (Sigma Aldrich, A5441), anti-CD63 (Thermo Fisher, 10628D), anti-TSG101 (Abcam, ab125011), and anti-calnexin (Abcam, ab22595). The following secondary antibodies were purchased from Invitrogen: Goat anti-Mouse IgG (H+L) Highly Cross-Adsorbed Secondary Antibody, HRP (A16078) and Goat anti-Rabbit IgG (H+L) Highly Cross-Adsorbed Secondary Antibody, HRP (A16110).

### Immuno-Transmission electron microscopy (Immuno-TEM)

Carbon film coated 200 mesh copper EM grids were soaked in 50 µL EVs (1x10^7^ Naïve, p88 and pep1 EVs in PBS) for 30 min for the adsorption of EVs on the grid. EVs on the grids were fixed by treating with 50 µL of 2% Paraformaldehyde (PFA) for 5 min and then rinsed thrice with 100 μL PBS. To quench free aldehyde groups, the grids were treated with 50 μL of 0.05 M glycine for 10 min. The surface of the grids was blocked with a drop of blocking buffer (PBS containing 1% BSA) for 30 min. After blocking, the grids were incubated with 50-100 μL anti-HA (Sigma-Aldrich H3663) or anti-CD63 (Thermo Fisher 10628D) antibody (1:100 in PBS containing 0.1% BSA) for 1 h. The grids were washed five times with 50 μL PBS containing 0.1% BSA for 10 min each. For secondary antibody treatment, the grids were incubated in a drop of Goat-anti-Mouse IgG coupled with 10 nm gold nanoparticles (Electron Microscopy Sciences, 25512) diluted at 1:100 in PBS containing 0.1% BSA for 1 h. The grids were washed five times with 50 μL PBS containing 0.1% BSA for 10 min each and then with two separate drops of (50 μL) distilled water. EVs were negatively stained with 2% uranyl acetate and then rinsed with PBS. The grids were then air dried for 24-48 h and images were captured by Transmission electron microscope (JEOL 1400) at 80 kV.

### Confocal Microscopy

2.5x10^6^ naïve, mCherry labeled, and p88-mcherry co-labeled EVs were loaded on a coverslip at three different locations. Dried and mounted EVs were then analyzed under a fluorescence microscope (Nikon Eclipse Ts2R) at 60X magnification. NIT-1 cells were cultured until passage 18 to ensure positive labeling with insulin 61. For EV binding studies, cocultured NIT-1 cells and 4T1 (3X10^4^) cells in a 4-well chamber slide (Nunc Lab-Tek) were treated with 1x10^7^ mCherry-EVs or mCherry co-labeled p88-EVs for 1 h at room temperature. Cells were rinsed with 1X PBS containing 0.1% Tween 20 to remove unbound excess EVs. To fix the cells, the slide was treated with 250 µL 4% PFA at room temperature for 10 min. The cells were washed thrice with ice-cold PBS, and treated with blocking buffer (1% BSA, 22.52 mg/mL glycine in PBST (PBS+ 0.1% Tween 20)) for 30 min. Then they were incubated in a humidified chamber with 250µL anti-insulin antibody (Guinea pig polyclonal Insulin antibody, Abcam ab7842; 1:100 diluted 1% BSA in PBST) overnight at 4°C. To remove unbound antibodies, the cells was washed thrice with PBS for 5 min each. Then they were treated with 250 µL solution of secondary antibody (Goat Anti-Guinea pig IgG H&L (FITC), Abcam ab6904; 1:1000 diluted in 1% BSA in PBST) for 1 h at room temperature in dark. After removing secondary antibody solution, the cells were washed thrice with PBS for 5 min each in dark. For counter staining, the cells were treated with 250 µL of 0.5 µg/mL solution of DAPI for 1 min. The cells were again rinsed with 1X PBS, and then coverslip was mounted on the cells after applying mounting medium (Life Technologies, P36930). The slide was stored at 4°C in the dark for further analysis of the cells using a confocal fluorescence microscope (Nikon A1Rsi).

### *In vitro* Bioluminescent assay

In this assay, naïve EVs, NP-gLuc EVs and p88-gLuc EVs were placed in wells of a 96 well plate (UV-Star® Microplate, 96 well, COC, F-Bottom (Chimney Well), uClear®, Clear; Greiner Bio-one) in triplicate. 95 µL of DPBS was added to each well and then treated with 50 µL 1 µg/mL Coelenterazine-H (CTZ; Regis Technologies). The luminescence was recorded using an *in vivo* imaging system (IVIS; Spectrum Perkin Elmer). For control 5µL of DPBS was used and treated in the same manner.

### *In vitro* Bioluminescent assay

The EV-binding assay was performed using bioluminescent imaging (BLI). NIT-1 mouse β-cell mouse pancreatic β-cells and 4T1 mouse mammary carcinoma cells were seeded at 1.0x10^4^ cells/96-well plates 24 h prior to EV treatment. The cells were fixed with 4% PFA at room temperature for 10 min and washed three times with PBS. The cells were treated with 1.0x10^7^ NP-gLuc-EVs or p88-gLuc-EVs in 100 µL media for 0, 30 and 60 min at 37 °C. Following the two PBS washes to remove unbound EVs, CTZ (1 µg/mL in PBS) was added to the wells and imaged by IVIS. Total photon flux (photons/sec) was quantified using Living Image 4.7.2 software (IVIS, PerkinElmer). Values are presented as the means ± SD (n = 3).

### Animals

In this study, female *Balb/cJ* mice (6 weeks old) were used for animal experiments. Animals were purchased from Jackson Laboratories and housed in the University Laboratory Animal Resources Facility at Michigan State University. All the experimental procedures for the animal study were performed with the approval of the Institutional Animal Care and Use Committee of Michigan State University.

### *Ex vivo* imaging of mice organs

Anesthetized mice received intravenously injection of 1.0x10^10^ NP-gLuc EVs, p88-gLuc EVs or PBS (n=2). Following 30 min circulation, the mice were sacrificed and the following visceral organs were dissected and placed on a transparent sheet: heart, lungs, liver, kidneys, pancreas and spleen. *Ex vivo* images of BLI were taken following re-application of 200 µL CTZ (10 µg) to the resected organs by IVIS. The images were quantified using Living Image 4.5 software (IVIS, PerkinElmer).

### Plasmid DNA Recovery from Animal Tissue

Approximately 1.0x10^9^ NP-EVs (n=2) or p88-EVs (n=3) in 100 µL PBS were intravenously injected into mice. Following 1 h of EVs administration, the mice were sacrificed, and the visceral organs (heart, lung, liver, kidney, pancreas and spleen) were dissected and homogenized using Triple-Pure High Impact 2.8mm Steel Beads (Benchmark Scientific) and BeadBug 6 Microtube Homogenizer (Benchmark Scientific). The plasmid DNA was isolated from the organ homogenates using QIAprep Spin Miniprep Kit following a modified protocol for plasmid isolation from mammalian cells that would exclude chromosomal DNA 62. The copy number of the plasmids was assessed by qPCR-based TaqMan assay.

### DNase I Treatment

The 2 µL of Naïve EVs mixed with 10^7^ copies of pDNA or p88-EVs were incubated at room temperature for 15 min with 1 U of DNase I (Zymo Research) and DNA Digestion Buffer. The plasmid DNA was isolated from the EVs using Qiamp Miniprep kits and quantified by qPCR.

### Quantitative Real-time Polymerase Chain Reaction (qPCR)

#### Evaluation of Plasmid DNA associated with EVs

qPCR was performed using Taq DNA polymerase (Fisher BioReagents). Each reaction contains 200 µM dNTP, 500 nM each of forward/reverse primer, 400 nM probe (Table S1), 0.5 U Taq DNA polymerase, 1x Assay buffer A and 1 µL sample DNA or isolated EV in a total reaction volume of 10 µL using CFX96 Touch Real-Time PCR Detection System (BIO-RAD). The PCR amplification cycle was as follows: 95°C for 2 min; 40 cycles of 95°C for 20 seconds, 65°C for 30 seconds. The pDNA copy number were determined by absolute quantification using the standard curve method, and the copy number of EV encapsulated pDNA per vesicles was calculated based on NTA and qPCR results.

#### Small DNA Recovered from each Organ

qPCR was performed using PrimeTime® Gene Expression Master Mix (Integrated DNA Technologies, Inc.). Each reaction contains, 500 nM each of forward/reverse primers, 200 nM probe (Table S1), and 1 µL sample DNA in a total reaction volume of 10 µL using CFX96 Touch Real-Time PCR Detection System (BIO-RAD). The PCR amplification cycle was as follows: 95°C for 2 min; 40 cycles of 95°C for 15 seconds, 65°C for 30 seconds. The recovered pDNA from each organ was normalized by recovered Mitochondria DNA.

#### Mitochondria DNA detection

qPCR was performed using Phusion® High-Fidelity DNA Polymerase (New England Biolabs, inc.). Each reaction contains, 0.2 U Phusion® High-Fidelity DNA Polymerase, 1x Phusion HF buffer, 200 uM dNTP, 1/20,000 diluted SYBR™ Green I (Invitorogen), 500 nM each of forward/reverse primer (Table S1), and 1 µL sample DNA in a total reaction volume of 10 µL using CFX96 Touch Real-Time PCR Detection System (BIO-RAD). The PCR amplification cycle was as follows: 98°C for 2 min; 40 cycles of 98°C for 10 seconds, 60°C for 20 seconds, 72°C for 20 seconds. The size of PCR product was analyzed by agarose gel electrophoresis.

## Results

### Design, Generation and characterization of engineered EVs displaying pancreatic β-cell targeting peptide

**Figure 1:**
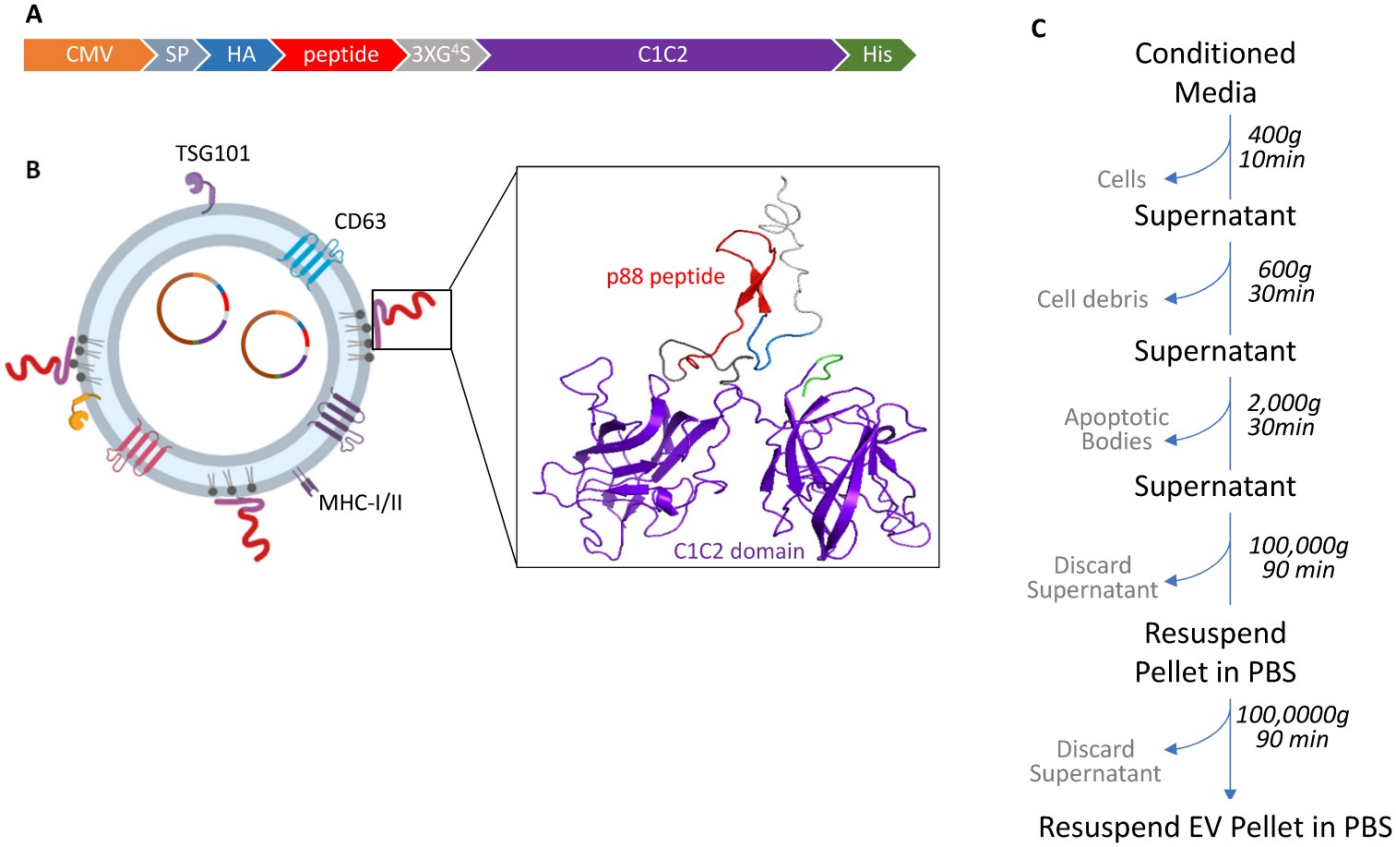
** Design and schematic presentation of a C1C2-peptide fusion protein (A)** Peptide-C1C2 fusion protein expression driven by a CMV promoter in pcDNA6.0 derived pcS vector. The recombinant protein comprising a lactadherin signal peptide (SP), Hemagglutinin tag (HA), peptide sequence, (GGGGS)_3_ Linker, EV anchor region of lactadherin (C1C2), and polyhistidine tag (HA). **(B)** Depiction of EV with engineered EV displaying peptide on its surface and encapsulated pDNA, and predicted protein structure of peptide-hC1C2 chimeric protein. **(C)** Schematic flow of EV isolation process.

We investigated whether EVs can display β-cell-targeting peptides on their surface to improve specificity and enhance EV-mediated cargo delivery to the target cells after systemic administration in living animals. The p88 peptide, known to bind (FXYD2)γa on pancreatic β-cells, was used as a targeting ligand for pancreatic β-cells 48. The pep1 peptide raised against p16-overexpressing cancer cells served as a control peptide with a negligible affinity for pancreatic cells in our system 63. In order to label EVs with these peptides, we fused peptide sequences followed by the (GGGGS)_3_ linker with the C1C2 domain of lactadherin (Fig. 1A and 1B). Following transfection, the EV fraction was collected from the culture media by ultracentrifugation, as described in Figure 1C.

**Figure 2:**
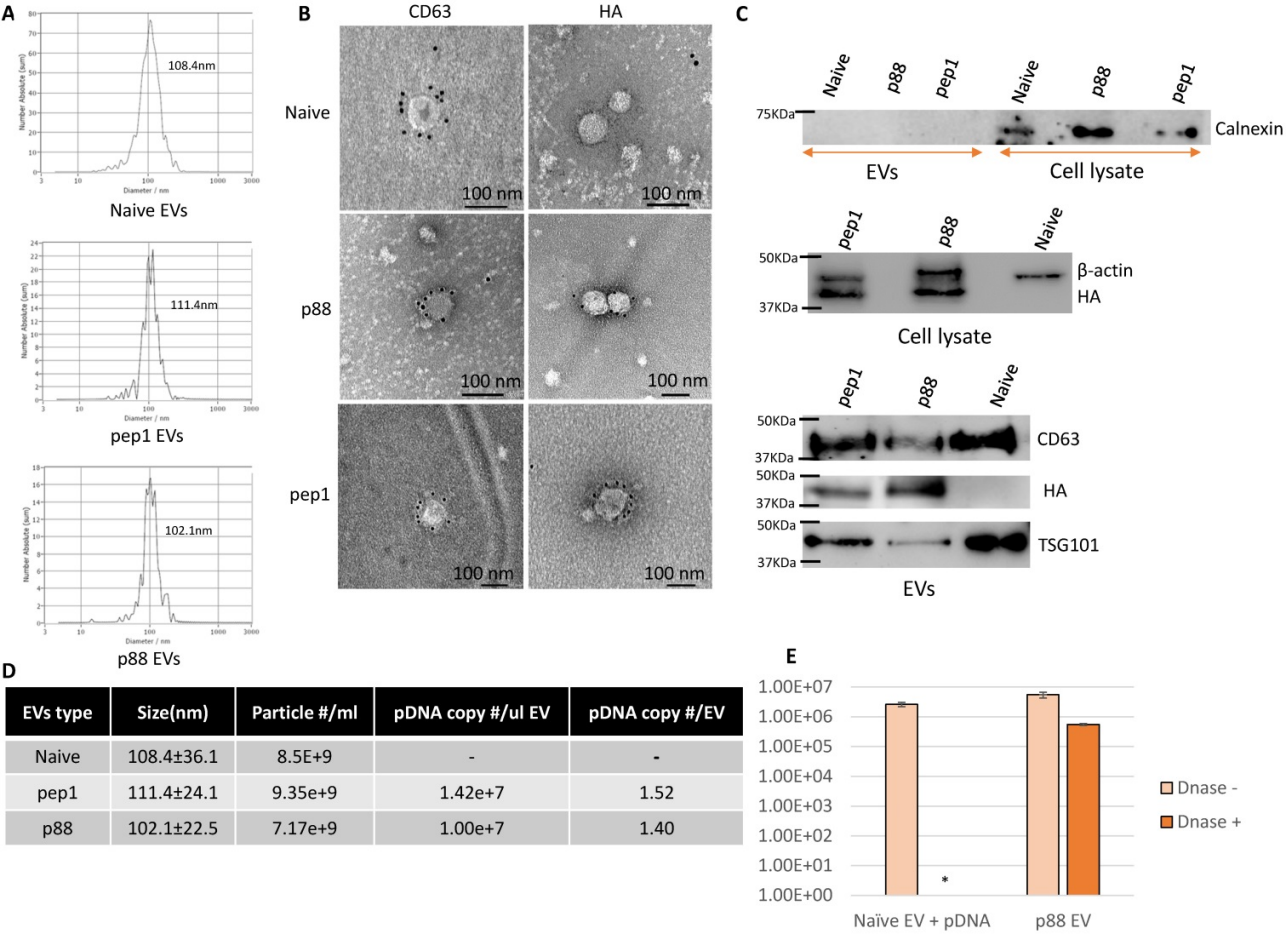
** Successful isolation and characterization of engineered EVs displaying peptides of interest. (A)** Representative size distribution of the naïve, pep1- and p88-display EVs determined by Nanoparticle Tracking Analysis. **(B)** Transmission electron microscopy images of naïve, pep1-, and p88-EVs showing gold labeled HA and CD63 surface markers.** (C)** Western blot analysis of engineered EVs (p88 fusion peptide-44Kda; pep1 fusion peptide-42KDa) for the presence of EV biomarkers CD63(30-60KDa) and TSG101(44KDa), and peptide HA-tag. Additionally, analysis of cell lysate and engineered EVs for cellular biomarkers Calnexin (67KDa) and β-actin (42KDa) **(D)** Summary of particle number and pDNA copy numbers determined by NTA and qPCR of pep1- and p88-display EVs. **(E)** pDNA quantification before and after DNase I treatment of EVs. Naïve EVs mixed with pDNA and p88 EVs were treated with DNase I. pDNA were quantified by qPCR following pDNA isolation. *pDNA undetected

In addition to being devoid of disease-related molecules, the engineered EVs generated from 293T cells projected minimal toxicity and immunogenicity effects in mice 64, 65. These EVs were characterized following MISEV (Minimal Information for Studies of Extracellular Vesicles 2018) guidelines 66. The particle size of 293T-derived EVs peaks around 100 nm at the concentration ranges from 1-9 x10^9^ particles/mL (Fig. 2A). The presence of peptide-C1C2 did not affect EV size or morphology (Fig. 2A and 2B). Immuno-TEM images demonstrated typical EV morphology with the presence of EV marker (CD63) and peptide (HA) on the engineered EVs (Fig. 2B and S1). As shown in Figure 2C, both p88-C1C2 and pep1-C1C2 fusion proteins were successfully expressed in these cells and on EVs, which appeared as bands proximal to their calculated molecular weights of 43.9 kDa and 42.0 kDa, respectively. Furthermore, all the EV types were positive for EV markers CD63 and TSG101, whereas cell-specific protein calnexin was not detected, further confirming the purity of the EVs without cell contamination. qPCR analysis using a probe specific to the encapsulated pDNA in combination with nanoparticle tracking analysis determined the quantity of pDNA in these vesicles. Notably, reducing pDNA size by removing non-essential sequences improved transfection efficiency and subsequent pDNA packaging efficacy (data not shown). Figure 2D shows representative data of engineered EVs analyzed by NTA particle count and qPCR data generated by a primer and probe set over plasmid specific regions. There were copy number variabilities in each batch of EVs, but the results consistently showed 0.3-2 pDNA copies per EV. Further, we examined the number of plasmids encapsulated inside of the engineered vesicles by EV-DNaseI treatment. As shown in Figure 2E, it removed approximately 90% of the plasmids, indicating that 10% of the total plasmid were encapsulated. Thus, the engineered EVs incorporate the majority of the DNA on their surface.

### Binding of targeting EVs to NIT-1 pancreatic β-cells *in vitro*

**Figure 3:**
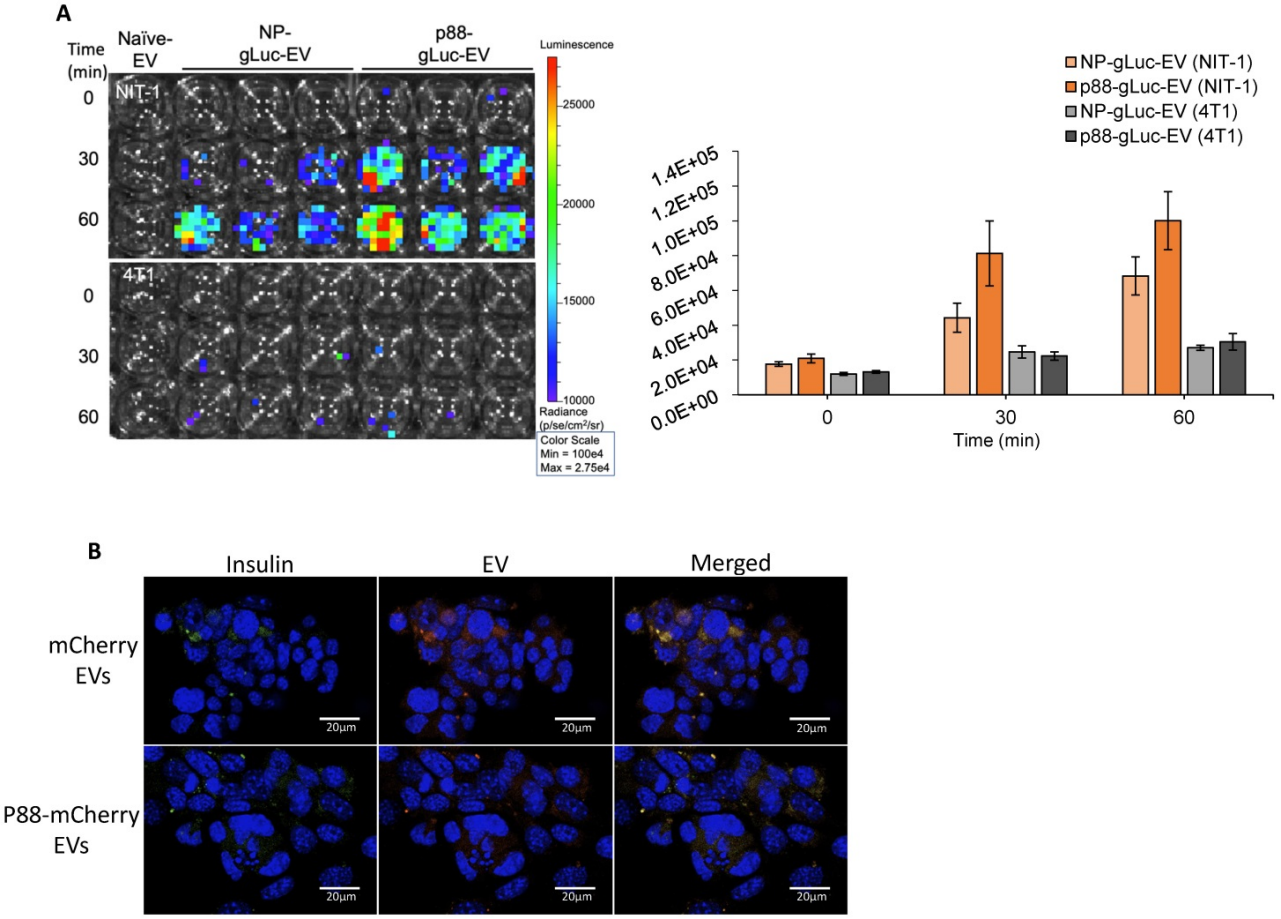
** Specific binding of targeting EVs to pancreatic β-cell line *in vitro*. (A)** NIT-1 cells and 4T1 cells received non-tarting (NP)- or β-cell-targeting (p88)-EV treatment after PFA fixation. Representative image of EV (gLuc) binding to NIT-1 or 4T1 cells. The total photon flu (p/s) from EVs bound to the cells was quantified using IVS. The value represents the means ± SD (n=3) in the graph. **(B)** NIT-1 and 4T1 cells were co-cultured and treated either with mCherry-EVs (upper row), or p88-mCherry-EVs (lower row) for 1 hr. The cells were fixed, and the binding was assessed by confocal imaging of EVs (red), anti-insulin antibody (FITC-conjugated) and nuclear staining with DAPI. microscopic images of cocultured NIT1 (FITC insulin labeled; Green), 4T1 cells treated with p88-mCherry-EVs, and mCherry-EVs (red). DAPI stain (blue) Scale bars, 20 μm.

GLuc labeled targeting (p88) and non-targeting (NP) (Fig. S2) were evaluated on the cultured cells using bioluminescence imaging (BLI) to examine the binding capacity of β-cell targeting EVs. NIT-1 mouse pancreatic β-cells and 4T1 mouse mammary carcinoma cells were treated with p88- or NP-EVs following PFA fixation. NIT-1 cells showed stronger bioluminescence after 30 min incubation with p88-gLuc-EVs, compared with NP-gLuc-EV-treated cells, demonstrating a higher binding capacity of p88-EVs towards β-cells compared to Non-peptide-EVs (Fig. 3A). There were very few signals detected for non-β (4T1) cells treated neither with p88- nor NP-EVs. To further evaluate the binding capacity towards β-cells, binding was observed using NIT-1 cells co-cultured with 4T1 cells.

EVs were labelled with mCherry (mCherry-EVs) or co-labelled with p88 and mCherry (p88-mCherry EVs) (Fig S3) prior to the treatment. After 1 h of incubation, the p88-mCherry-EVs (red) mainly localized to the insulin-secreting β-cells (green). In contrast, the EVs without p88 (red) bound non-specifically regardless of cell type (Fig. 3B), indicating peptide mediated EV binding to the β-cells. Time-course experiments using NIT-1 cells to determine pDNA uptake using p88-EV and pep1-EV showed that the rate of pDNA uptake was not distinguishable between two EV types (Fig. S4A). The further analysis using the non-peptide (NP-EVs) version of the construct showed similar trends (Fig. S4B), suggesting the limitation of the DNA uptake assay with the static and restricted flow of the tissue-culture cellular environment. The uptake usually starts as early as 10 min for targeting EVs and saturate at 1 h time point for both non-targeting and targeting EVs.

### Biodistribution of pancreas targeting by engineered EVs

**Figure 4:**
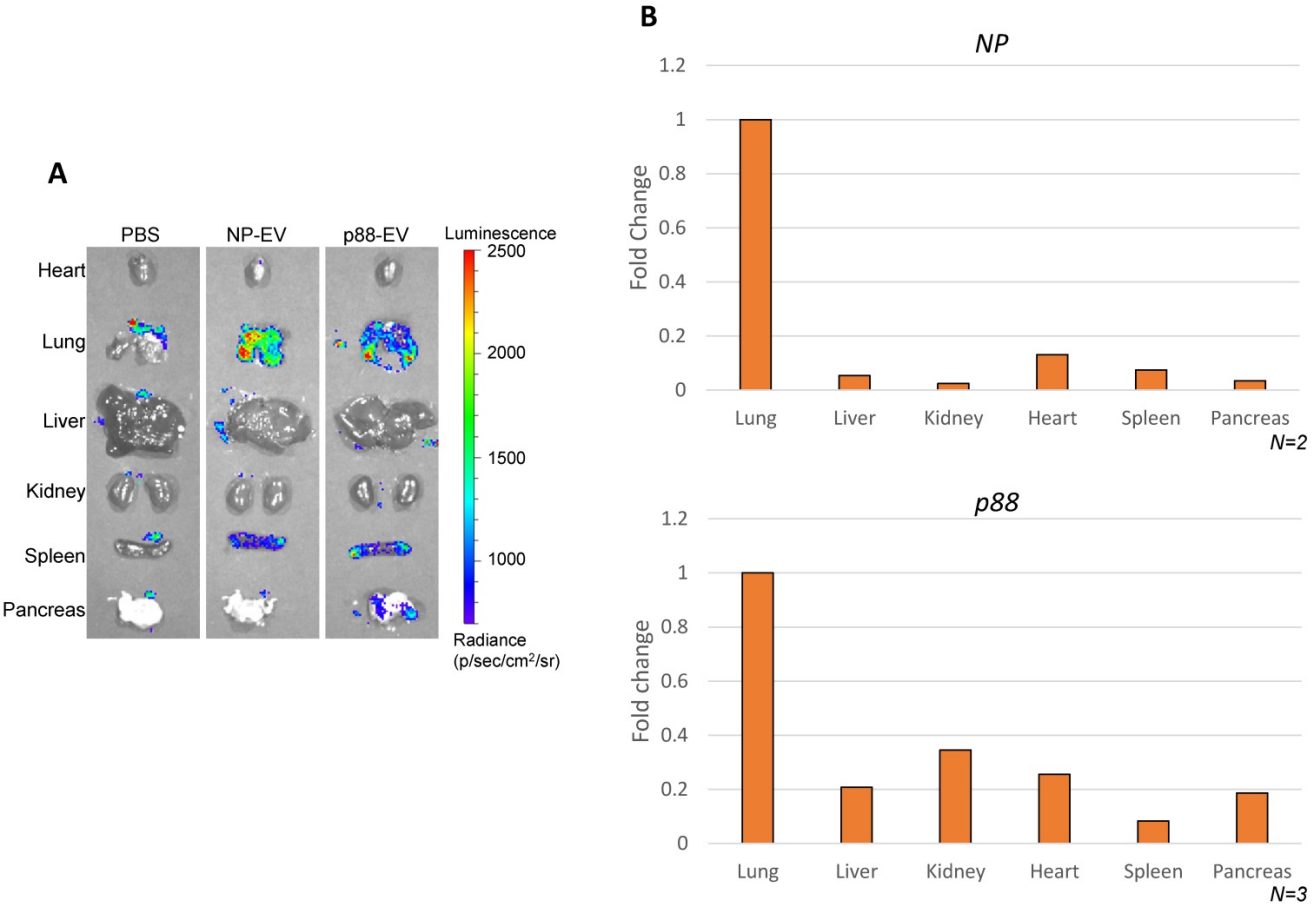
** Altered EV biodistribution by peptide-display, and pancreas enriched pDNA delivery by β**-**cell-targeting EVs. (A)** Representative images of the organs from the Balb/cJ mice received intravenous injections of PBS, NP-gLuc- or p88-gLuc-EVs. **(B)** Balb/cJ mice received intravenous injection of NP- or p88-labeled EVs. The organs were removed from the mice post-mortem and homogenized for pDNA isolation. qPCR assay was used to determine the copy number from heart, lungs, liver, kidneys, pancreas and spleen. The amounts of recovered pDNA were normalized by mitochondrial DNA. The fold change values represent the average fold change of samples (N).

Biodistribution of targeting (p88-gLuc) and non-targeting (NP-gLuc) EVs in live mice were evaluated by *ex vivo* imaging. A high background signal of the substrate was captured in the control mice organs treated with PBS (Fig. 4A), which was indistinguishable from the signals received from mice treated with engineered EVs as previously reported 67. The signals from the internal organs of the respective mice (Fig. 4A and S5A) verified the circulation of EVs in the body. The signals from the non-targeting EVs were primarily detected in the lung and spleen. The bioluminescence signals representing targeted EVs were predominantly observed from the lung, spleen and pancreas, implying the affinity-mediated navigation of EVs to the pancreas following the typical circulation pattern through the lungs and heart. The background luminescence of the substrate was spotted only in the lungs of control mice, and faded upon dilution in blood circulation by heart, as no signal was detected in other organs. The experiment verified directed steering of targeting EVs by the displayed peptide to the pancreas through systemic blood circulation.

### EV-mediated targeted delivery of pDNA *in vivo*

The ability of p88-EVs to selectively deliver pDNA *in vivo* was assessed by recovering and quantifying pDNA from pancreatic homogenates of mice 1 h post-administration with targeting EVs. pDNA recovery from organs were confirmed by the mitochondrial DNA (mtDNA) qPCR (Table S2). Strikingly, the pancreas of mice that received pancreas targeting p88-EVs exhibited the accumulation of p88-coding pDNA in contrast to the spleen, as shown in Figure 4B. We further demonstrated that assayed pDNA are indeed plasmid and not fragmented DNA pieces by qPCR analysis using the primer sets over 2 other regions of the plasmid (ampicillin gene and C1C2-coding region). All of the qPCR assays used in this work uses primer sets amplifying the peptide-coding region of the EV-display plasmid (Fig. S5B).

## Discussion

There is significant research interest in developing EV therapeutics that harness the innate ability of EVs to mediate the delivery of a diverse selection of cargos 9, 10, 12, 23-25, 32-34, 68. Unlike synthetic nanocarriers, EVs feature more robust stability *in vivo* since they do not provoke strong immunogenic responses or toxic side effects 1. Furthermore, EVs can be engineered with relative ease by fusing targeting moieties to known EV surface proteins like Lamp2b, tetraspanins (CD63, CD81, CD53, CD37, and CD82), and Lactadherin 37, 39-41, 69. In this work, we demonstrated targeted delivery of pDNA to the pancreas using engineered EVs that display a β-cell specific peptide* in vivo*. We also analyzed the EV targeting by *in vivo* bioluminescence imaging and exogenous EV-mediated delivery by quantifying the pDNA copy number within engineered EVs and resected organs.

Through the surface display of the p88 peptide, the engineered EVs attained affinity towards the ion transport regulator (FXYD2)γa displayed on pancreatic islets 48, 49, 70. The p88 peptide, in combination with a Gadolinium-based contrast agent, was previously developed for non-invasive *in vivo* imaging and quantification of β-cells in MRI studies 48, 49. We showed EVs engineered to display p88 (p88-EVs) exhibited higher binding capacity to β-cells in co-cultured cells *in vitro* (Fig. 3B). The *in vitro* DNA uptake study showed high variabilities indicated by the large error bars, suggesting the limitation of the *in vitro* assay (Fig. S4A, B). This could be due to the restricted cultured environment cause uptake and release of exogenous pDNA in the simultaneous manner. Thus, the *in vitro* assay does not represent the biological response *in vivo* and is not conclusive.

Molecular display using lactadherin C1C2 fusion proteins was used to improve EV targeting in several studies 22, 42, 71-73. While the exact mechanism of C1C2 localization to EV membranes still remains unclear, the use of C1C2 fusion proteins, when bound to PS on EV membrane surfaces, provides additional benefits such as inhibiting the recognition of PS by coagulation factors and macrophages 43, 44. Thus, purified C1C2-fusion proteins can be reconstituted with isolated EVs to engineer the surface of EVs in a plug-and-play manner 42, 73.

Pancreatic β cells are relatively inaccessible as a drug target due to their deep anatomical location and structure on the islet. This limits diagnostic and therapeutic options. Previous attempts to target the pancreas, for example apratoxin A, showed high antiproliferative capabilities, though it caused notable *in vivo* toxicity 50. We show that *in vivo* systemic administration of β-cell targeting EV harnessed targeting delivery of cargo pDNA to the pancreatic cells (Fig. 4B). Importantly, to our knowledge, this is the first study to show the recovery and quantification of exogenously introduced pDNA cargo to the pancreas, whereas previous studies demonstrated the physiological changes without clearly determining the type of molecules (DNA/RNA/protein) delivered to their target cells 30, 39, 74, 75. The efficient DNA recovery was confirmed by co-isolation of high-quantity mitochondrial DNA (Table S2).

While (FXYD2)γa, which was used in this study, has been identified as a specific biomarker to pancreatic β-cells, its expression is not limited to the pancreas. This ion transport regulator has been found in the liver, kidney, salivary gland and gallbladder of humans, in addition to the pneumocytes of macaques 48, 49, 70, 76. This could possibly explain pDNA delivery by p88-EVs to non-target organs (Fig. S4), but the total number of copies of pDNA delivered to the pancreas remains significantly higher compared other organs (Fig. 4C). Although the p88-pDNA recovery from the pancreas was consistently high from tested animals, the pDNA copy number has variabilities between animals and among organs, and at 2000 copies per pancreas, efficiency could be improved. The variability could be due to circulation time, sample processing and variabilities between EVs quality from the storage durations and conditions, which require improvements.

The confocal microscopic imaging of the EVs in co-cultured cells clearly demonstrated that p88-EVs binds to pancreatic β-cells in culture within 1 h (Fig. 3B). EVs were labeled with mCherry using endogenous mCherry-C1C2 pDNA transfection method, and fluorescence was verified prior to treatment (Fig. S3).

*In vivo* EV imaging was also used to demonstrate targeting, but their nanosized and labeling limitations complicate EV visualization. For example, fluorescent lipophilic dyes (PKH67, PKH26, R18, Dil, and DiD) stain the EV membrane, but only allow for imaging after harvesting the organ due to its low sensitivity 36, 77-79. Furthermore, lipophilic dyes persist in tissues or circulation even after the degradation or dissociation of EV's; so, in the case of using lipophilic dyes, EVs must be differentiated from the dye itself and additional background signals 36, 69, 80, 81. Radiolabeling of EVs, using ^99m^Tc, ^131^I, and ^111^In-oxine, are promising in non-invasive biodistribution studies, offering both quantitative and qualitative data 80, 82, 83. However, there are still some limitations to overcome, such as short half-life and inefficient EV recovery post labeling. In this study, the luciferase from *Gaussia princeps*, a reporter protein that emits bioluminescence in the presence of its substrate coelenterazine, was used; this was similar to the approach described by Takahashi et al 58. In line with their findings of EV monitoring in live mice, we observed a similar pattern of diffuse signals over the ventral side of mice and absence of any localized signal. However, our bioluminescent signals did not extend up to 30 mins post substrate injection. Although, we used similar bolus i.v. injection of the substrate, we only detected signals until 10-15 mins post substrate injection. In addition to potential nonspecific interference from vascular epithelial cells or blood cells, the presence of FXYD2 receptors in other tissues such as salivary and mammary glands, dorsal root ganglia, and kidney could cause the observed signal scattering.

The importance of EV-mediated DNA delivery lies in the potential of directed transfer of therapeutic DNA *in vivo*. The methods used in our work combined simple well-established methods to show targeted pDNA delivery, including qPCR DNA quantification from EVs and the harvested organ. Alternatively, next-generation sequencing to identify and quantify all RNA biotypes or shotgun proteomics could have been used to provide broad-scale data analysis 79, 84. Targeting is key to effective delivery of therapeutics allowing precise localization to diseased tissues and thus eliminating side effects derived from off-target effects of large drug dosage.

There are still inefficient and variable loading and DNA into EVs and recovery among sets of experiments, but our data holds promise for future customized therapeutics. Precise targeting may allow for guided-tissue regeneration by delivering genes to stem cells in targeted tissues. Similarly, this approach could deliver genes to create producer cells in target tissues to generate bystander effects which can influence groups of surrounding cells as was reported by Kanada et al 28. As mentioned, efficient gene packaging and generation are limitations yet to be overcome for effective EV-mediated DNA delivery. EV generator cells with engineered surface ligands cloned into the genome may allow efficient EV production at scale. Electrical stimulation of cells (live cellular nanoporation) to efficiently package mRNA to EVs and to increase EV production would increase EV numbers from producer cells 85. The combinations of such cutting-edge technologies will shed lights to targeted EV therapeutic, especially for diseases with no existing treatment.

This report adds to a growing body of literature demonstrating the potential - and shortcomings - of targeted EV Therapeutics. We believe that future improvements in targeting specificity, DNA packaging and EV isolation would open new perspectives into EV-mediated therapeutic delivery as a safe and effective technology.

## Conclusion

In conclusion, our engineered EV generation technique is simple, robust, and efficient. This study demonstrates that small peptide-based ligands can impart affinity to EVs upon being displayed on the surface. EV-mediated targeted delivery was achieved without any observed toxicity in the cell lines or visible side effects on the mice. We believe that the EV-mediated targeted delivery can be leveraged for treating human pancreatic diseases. Moreover, conjugating therapeutic molecules/drugs/imaging probes with engineered EVs can be applied for investigating targeted delivery in other clinically significant organs. To tap into the tremendous potential of EV-mediated targeted delivery, further *in-vivo* research is needed to improve the pharmacokinetic profile of delivery systems and minimizing non-specific uptake of EVs.

## Supplementary Material

Supplementary figures and tables.
