# Engineering Extracellular Vesicles to Target Pancreatic Tissue *In Vivo*: Erratum

**DOI:** 10.7150/ntno.75006

**Published:** 2023-01-01

**Authors:** Hiroaki Komuro, Yuki Kawai-Harada, Shakhlo Aminova, Nathaniel Pascual, Anshu Malik, Christopher H. Contag, Masako Harada

**Affiliations:** 1 Department of Cardiovascular Medicine, Tokyo Medical and Dental University, Tokyo, Japan.; 2 Institute for Quantitative Health Science and Engineering (IQ), Michigan State University, Michigan, USA.; 3 Lyman Briggs College, Michigan State University, Michigan, USA.; 4 Department of Chemical Engineering and Material, Michigan State University, Michigan, USA.; 5 Department of Microbiology and Molecular Genetics, Michigan State University, Michigan, USA.; 6 Department of Biomedical Engineering, Michigan State University, Michigan, USA.

In the original paper on p.380 and p.381, Material and Methods “Cell Culture and Treatment” section, there is an error in the construct used to label EVs for fluorescence. Our original paper incorrectly states that we used pcDNA-mCherry-C1C2 when it should have been pcS-mCherry-C1C2. The main differences between these two constructs are the backbone size, as pcS-mCherry-C1C2 is smaller by approximately 2kbp, without any changes in the protein-coding region.

In addition, on p.379 and p.380, Material and Methods “EV-peptide display plasmid construction” section, the pcDNA-mCherry-C1C2 was subjected to remove unnecessary sequences through the single-piece SLiCE reaction of the PCR fragments using the same method to pcS-p88-C1C2 or pcS-pep1-C1C2. These corrections do not change the main conclusions of this article.

