# Supplementary material for: Engineering Extracellular Vesicles to Target Pancreatic Tissue In Vivo
Source: Nanotheranostics. 2021 Apr 15;5(4):378–90. doi: 10.7150/ntno.54879 (PMC8077969; doi:10.7150/ntno.54879)
Supplement: Supplementary file 1 — Supplementary figures and tables. [file ntnov05p0378s1.pdf]

Supplemental Materials and Methods

In vitro cell uptake assay (pDNA)

NIT-1 cells were seeded at a density of 2.0×10^5 and 6.0×10^5 cells per well in 12-well and 6-well plates respectively 24 h prior to treatment with EVs. Cells were treated with 1.0×10^7 pep1-, NP- or p88-EVs for 10, 30, and 60 min in 1mL F12K medium supplemented with EV-depleted FBS. After incubation, the cells were washed twice with PBS and trypsin/EDTA treated for harvesting. DNA extraction from cell pellets was performed using QIAprep Minipre Kit, and the copy number of the plasmids was analyzed by qPCR.

qPCR was performed using Taq DNA polymerase (Fisher BioReagents). Each reaction contains 200 µM dNTP, 500 nM the forward/reverse primers, 400 nM probe (Table S1), 0.5 U Taq DNA polymerase, 1x Assay buffer A and 1 µL DNA sample or isolated EV in a total reaction volume of 10 µL using CFX96 Touch Real-Time PCR Detection System (BIO-RAD). The PCR amplification cycle was as follows: 95°C for 2 min; 40 cycles of 95°C for 20 seconds, 65°C for 30 seconds. The pDNA copy numbers were determined by absolute quantification using the standard curve method.

Supplemental Data

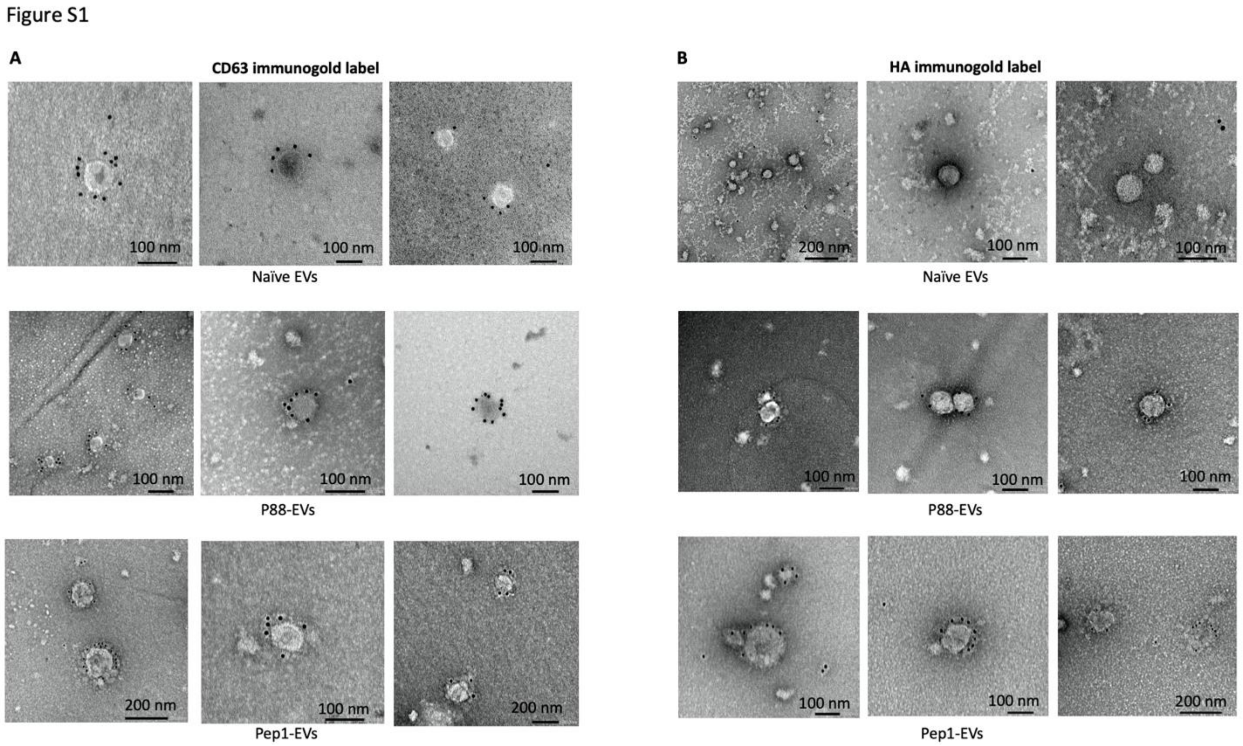

Figure S1. Transmission Electron microscopic images of **(A)** CD63 labeled and non-HA labeled Naïve EVs **(B)** CD63 and HA labeled p88-EVs(C) CD63 and HA labeled pep1-EVs.

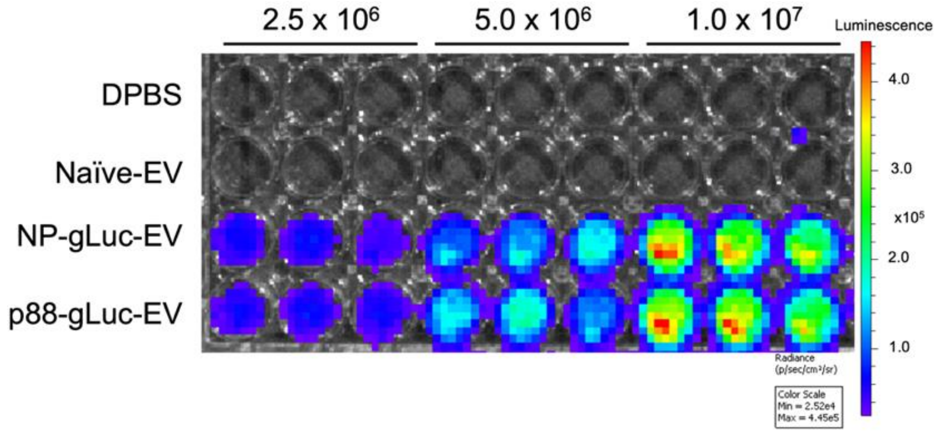

Figure S2. Fluorescence microscopic image of Naïve EVs, mCherry labeled EVs and p88+mcherry co-labeled EVs.

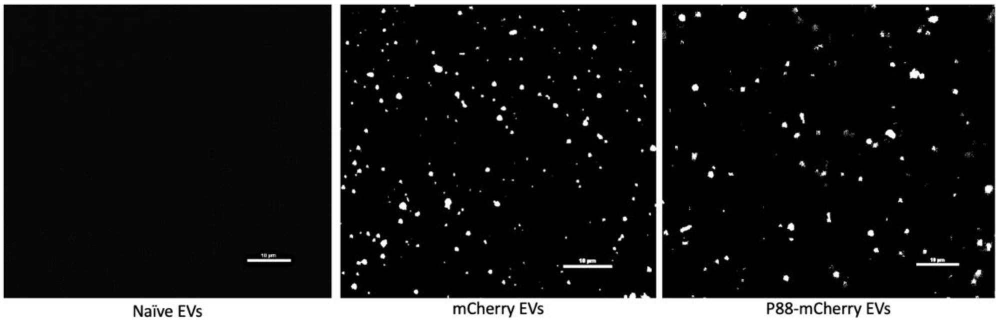

Figure S3. Bioluminescence radiance measurement of Naïve EVs, NP-gLuc EVs and p88-gLuc EVs.

A

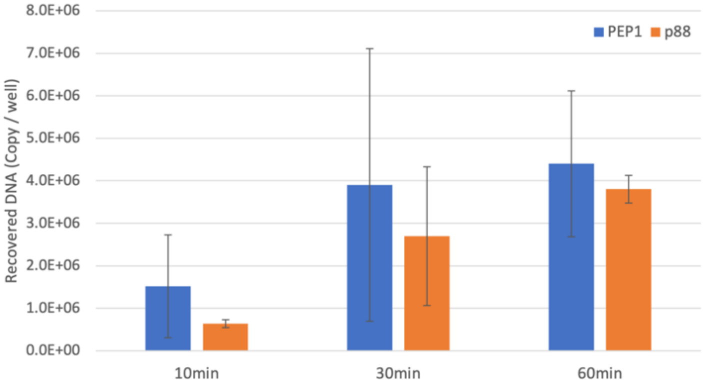

B

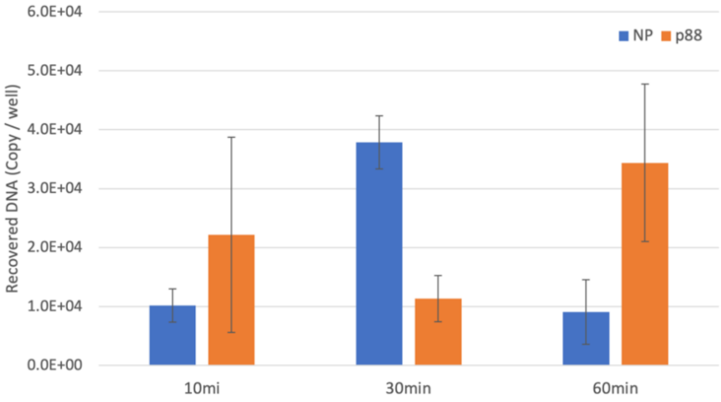

Figure S4. Fold change comparison of pDNA uptake using **(A)** Pep1- and p88 EVs or **(B)** NP- and p88-EVs by NIT-1 cells. The average changes were taken from sample duplicates of two independent experiments. Each set contains two technical duplicates.

A

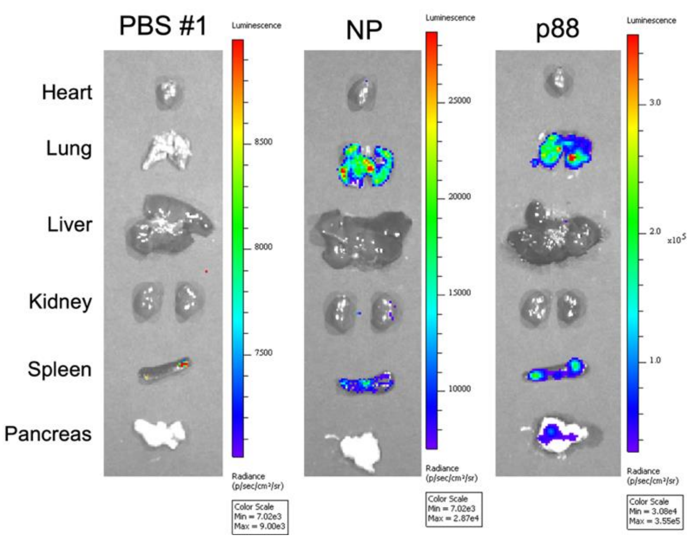

B

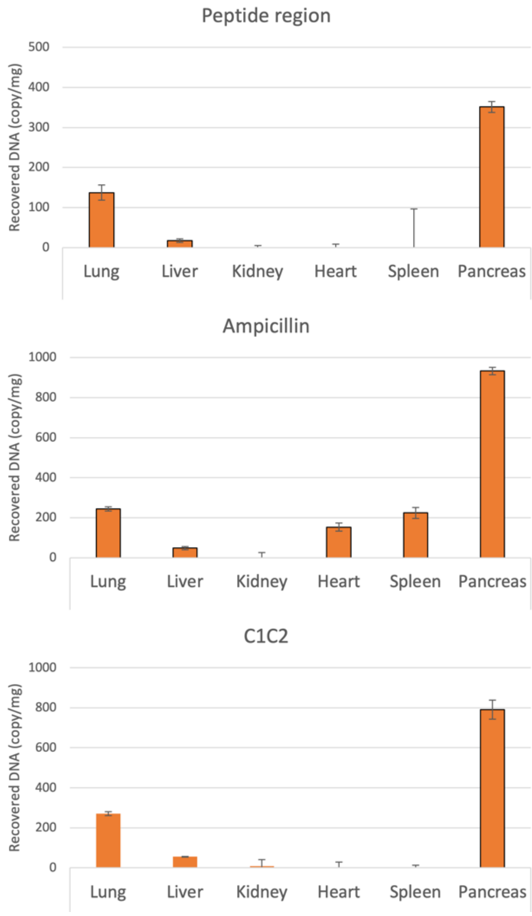

Figure S5. **A**) An additional set of ex vivo imaging following gluc-labeled EV administration, resection and CTZ application, revealing p88 EV accumulation in the pancreas **B**) qPCR analysis of plasmid fragments recovered from the organs of EV injected animals, determined by the primer sets, amplified the peptide-coding region, the ampicillin-coding region and the C1C2-coding region post 1h p88-EV injection. This confirms the plasmid DNA delivery, not the fragmented pDNA detection from the retrieved organs.

Table S1. Table of all the primers and oligonucleotides used in the study.

	Primer	Sequence (5'-3')	PCR Fragment (overhang)
1	pcD-ME8SS-F2	GAGACCCAAGCTGgctagcGTGTACAAAAAAGCAGGCACCATGCCGCgc	Lactadherin signal peptide
2	ME8SS-HA-R	agcataatctggaacatcatatggataGGCGACGAGGAGGCTGGGGGC	
3	HA-mCherry-F	atgatgttccagattatgctGTGAGCAAGGGCGAGGAGG	mCherry
4	mCherry-C1C2-R	tccatgcccagtggctcgacacatttCTTGTACAGCTCGTCCATGCCGC	
5	mCherry-C1C2-F	gcatggacgagctgtacaagAAATGTGTCGAGCCACTGGGCATGG	Lactadherin C1C2(mCherry)
6	hME8C1C2-pcD-R2	TGATGGTGATGATGACCGGTacagcccagcagctccaggcgc	
7	HA-gLuc-F3	atgatgttccagattatgctAAGCCCACCGAGAACAACGAAGAC	gLuc
8	gLuc-C1C2-R2	tccatgcccagtggctcgacacatttGTCACCACCGGCCCCCTTGATCT	
9	gLuc-C1C2 F	caagggggccggtggtgacAAATGTGTCGAGCCACTGGGCATGG	Lactadherin C1C2(gLuc)
10	hME8C1C2-pcD-R2	TGATGGTGATGATGACCGGTacagcccagcagctccaggcgc	
11	3x4GS-C1C2-BB_F	AATGTGTCGAGCCACTGGGC	pep1 backbone
12	pep1-HA-BB_R	AGCATAATCTGGAACATCATATGGATAGGC	
13	3xG4S-F	GGTGGAGGCGGTTCAGGC	p88 backbone
14	pcDNAPEP1-HA-R	AGCATAATCTGGAACATCATATGGATAGGC	
15	pcU-BGHR-F	GGGGATAACGCAGGAAAGAAC	pcS primer sets
16	pcU-BGHR-R3	TCCTGCGTTATCCCCCCATAGAGCCCACCGCAT	
17	qPCR Forward	GACCCAAGCTGGCTAGCGTGTAC	qPCR primer & probe
18	qPCR Reverse	GCCCAGTGGCTCGACACATTC	
19	qPCR probe	FAM/AGCCTCCTC/ZEN/GTCGCCTATCCATATGA/IBFQ	
20	HA-3xG4S_F	gttccagattatgct GGTGGAGGCGGTTCAGGC	pcS-NP-C1C2
21	HA-R	agcataatctggaacatcatatgg	
22	qPeptide_F	GACCCAAGCTGGCTAGCGTGTAC	qPCR primer & probe for peptide region
23	qPeptide_R	GCCCAGTGGCTCGACACATTC	
24	qPeptide_P	FAM/AGCCTCCTC/ZEN/GTCGCCTATCCATATGA/IBFQ	
25	qAmp-1_F	CTAGAGTAAGTAGTTCGCCAGTTAAT	qPCR primer & probe for ampicilin region
26	qAmp-1_R	GCTGAATGAAGCCATACCAAAC	
27	qAmp-1_P	ATTGCTACAGGCATCGTGGTGTCA	
28	qC1C2-1_F	CCTACAAGGTTGCCTACAGTAAT	qPCR primer & probe for C1C2 region
29	qC1C2-1_R	CTTCTTGTGGGAGTGGTTGT	
30	qC1C2-1_P	TGCCAGGGAAGATCTTACTGCTGC	
31	Mt16S_F	CCGCAAGGGAAAGATGAAAGAC	qPCR primer for mtDNA
32	Mt16S_R	TCGTTTGGTTTCGGGGTTTC	
Synthetic DNA fragments			
1	pep1-3xG4S-C1C2	GTGGCTCGACACATTCCGATCCGCCACCGCCAGAGCCACCTCCGCCTGAACCGCCTCCACCATGATGCAGC AGGCTATGGCTAGCATAATCTGGAAC	pep1-G4S fragment
2	P885-3-1	GTTCCAGATTATGCTCTGCCGCTGAGCCGCCATTATGGCGGCGGCAGCGTGCCGTTTTAT	p88 fragment
3	P885-3-2	AGCGTGCCGTTTTATAGCCATAGCAACACCCATCATACCAGCATGGGTGGAGGCGGTTCA	

Table S2. mtDNA value from 1/100 sample dilution indicating high recovery of plasmid DNA from each organ

EV-type	p88-EV	NP-EV
Lung	18.105	19.18
Liver	14.25	15.54
Kidney	16.68	16.08
Heart	17.18	19.21
Spleen	17.945	18.84
Pancreas	15.32	15.125
